# Our Contemporaries

**Published:** 1914-11

**Authors:** 


### OUR CONTEMPORARIES.

---

The Boston Medical and Surgical Journal, one of our two seniors in the western continent, in the issue of September 17, mentions two accidents due to speeding ambulances. One was not serious. The other resulted in the death of a boy from fracture of the skull and in an amputation of an arm for compound fracture of the elbow, of the ambulance chauffeur. The patient in the ambulance was being conveyed from another town and was not in a critical condition. Such instances are not uncommon. It is only occasionally that a case requires excessive haste, either on the part of the physician or of the ambulance. Whether, in any instance, the needs of the actual patient justify potential death and injury to others, is very doubtful. In the great majority of instances, violation of traffic ordinances by ambulances, physicians, fire officials and others, are not only without justification but are a subversion to private ends of an official privilege. For instance: a physician in private practice insisting on breaking through a parade line and bawling out to the policeman and the crowd, that he was Dr. So-and-so; a medical officer arrested for gross violation of traffic laws and released immediately on establishing his identity—no emergent case, no official duty, simply the desire to get into a good place to see a procession; another medical officer with his carburetor set so that his car would scarcely run under 25 miles an hour; a touring car bought by taxpayers for the police department, running at a rapid rate on the left of a turn—and no fire in progress; and ambulances running at excessive speeds without regard to halting for street cars or anything else, almost as a routine. Sometime, the humble tax-payer, liable to arrest and fine on any technical subterfuge when the pension fund gets low, will assert himself.

---

**Federal Licensure.** The Medical Council, September and November, 1914, contains editorials on the desirability and methods of obtaining federal licensure and asks for editorial comment generally. As to the constitutional difficulties in the way of any radical change in existing limitations of state and national authority and the practical means of overcoming them, we do not feel competent to express an opinion. As to the general desirability of general licensure of national scope, we express approval. Indeed, without attempting to speak for our readers or as expressing a definite policy of this journal, we are willing to express the personal editorial opinion that any general principle that is not materially influenced by local conditions, should be applicable throughout the country. We will go further and say that we think it is high time this country should be under one central government and that there should be under this government, only one other, that of the municipality or of some other strictly local unit, let us say the county. Possibly, there should be a further provision for small centers of concentrated population, having different problems from those of rural communities and not of sufficient size to warrant an independent charter outside of county boundaries. Before condemning this opinion as chimeric, it should be understood that it is not an original opinion but was held by many members of the constitutional convention who clearly expressed the expectation that the state governments would be continued only temporarily.

